# Effect of Fish Oil and Linseed Oil on Intake, Milk Yield and Milk Fatty Acid Profile in Goats

**DOI:** 10.3390/ani13132174

**Published:** 2023-07-01

**Authors:** Lam Phuoc Thanh, Juan J. Loor, Duong Tran Tuyet Mai, Tran Thi Thuy Hang

**Affiliations:** 1 Department of Animal Sciences, Can Tho University, Ninh Kieu, Can Tho 94000, Vietnam; 2 Department of Animal Sciences, Division of Nutritional Sciences, University of Illinois, Urbana, IL 61801, USA; 3 Department of Agricultural Technology, Can Tho University, Phung Hiep, Hau Giang 95000, Vietnam; tranthithuyhang@ctu.edu.vn

**Keywords:** dairy goats, intake, milk fatty acids, milk yield, rumen fermentation

## Abstract

**Simple Summary:**

Improvements in health-promoting milk fatty acids by feeding oil mixtures have been reported in dairy cows. However, in some cases, oil addition reduces milk yield and milk fat content. It is unknown whether the inclusion of linseed oil and fish oil at a high level in goat diets increases health-promoting fatty acids in milk without affecting milk production. The objective of this study was to investigate the effect of linseed oil added alone at 2.5% or in combination with tuna fish oil at 2.5% or 4.16% in goat diets on intake, milk yield, and milk fatty acid profiles. Compared with the control without oil addition, feeding linseed oil and fish oil at 4.16% markedly increased the levels of health-promoting fatty acids in milk such as *c*9,*t*11 conjugated linoleic acid, and docosahexaenoic acid but decreased milk total saturated fatty acids, atherogenicity, and thrombogenicity indices. Oil addition did not have a negative effect on intake, milk yield, and milk fat content. Thus, supplementing linseed oil and fish oil at 4.16% in the diet of lactating goats could have a positive impact on human health without any adverse effects on animal performance.

**Abstract:**

This study aimed to evaluate the effect of incorporating linseed oil and fish oil in the diet on intake, ruminal fermentation, milk yield, and milk fatty acid profiles in dairy goats. Four crossbred Saanen lactating goats in mid-lactation and milking 1.30 ± 0.28 g/day were used in a 4 × 4 Latin square design. The basal diet contained concentrate and Para grass (C:F 40:60). Treatments included a basal diet without oil supplementation (Ctrl) or with 2.5% linseed oil (LO_2.5_), 2.5% linseed oil and fish oil (3:2, *w*/*w*, LFO_2.5_), and 4.16% linseed oil and fish oil (3:2, *w*/*w*, LFO_4.16_). Diets had no effect on intake, milk yield, milk composition, or ruminal fermentation (*p* > 0.05). Compared with Ctrl, lower (*p* < 0.05) proportions of C10:0–C14:0 in milk fat were observed with LFO_4.16_. Compared with the Ctrl and linseed oil added alone, feeding LFO_4.16_ led to a greater (*p* < 0.01) concentration of C18:1 *t*11. Compared with both the Ctrl and LO_2.5_ diets, milk *c*9,*t*11 CLA was 4.53 and 2.94 times greater with the LFO_4.16_ diet. Compared with Ctrl and LO_2.5_ diets (0.06% and 0.08%), goats fed LFO_2.5,_ and LFO_4.16_ had greater (*p* < 0.001) concentrations of C22:6*n*-3 (0.63% and 0.87%). Overall, the combined data suggested that including 4.16% linseed oil and fish oil in the diet of dairy goats was effective in improving the concentrations of health-promoting fatty acids in milk without affecting milk production.

## 1. Introduction

In recent years, there has been a significant increase in milk consumption worldwide, largely due to the continuous rise in the global population. A previous report [1] revealed that between 2007 and 2017, the global goat population increased by 21.5%. Until now, Asia is ahead of other regions with the largest goat population, currently accounting for 56% of the world’s total. Furthermore, the rise in income and better living standards among people has led to a growing trend of consuming premium goat milk products in place of cow’s milk. The small size of milk fat particles, high digestibility, and low allergy potential are the main reasons goat milk is a preferred choice for infants [2].

The fat in goat milk contains a ratio of *n*-6 to *n*-3 fatty acids (FA) of 5:1, which is similar to the recommended ratio for the prevention of cardiovascular diseases in humans [3]. The potential advantages offered by goat milk products have resulted in an increase in their consumption by humans. Recent developments in dairy goat production have focused on enhancing the nutritional value of milk and dairy products as well as improving their functionality [4]. In order to improve the quantity and quality of milk, changes in the nutritional management of goats need to be made. For instance, goat milk produced through traditional dairy farming methods typically contains negligible amounts of conjugated linoleic acid (CLA), with α-linolenic acid (ALA) and docosahexaenoic acid (DHA) being barely detectable [5]. Previous research has indicated that adding linseed oil to the diet substantially increases the amount of unsaturated fatty acids (UFA) present in goat and cow milk, particularly CLA and ALA [6,7,8]. However, when linseed oil is added alone to the diet, it undergoes biohydrogenation (BH) by rumen microorganisms leading to the conversion of most of the ALA and CLA to stearic acid (SA, C18:0). This results in a substantial loss of ALA and CLA in milk.

In an in vitro study, the replacement of linseed oil with fish oil significantly elevated contents of ALA and DHA in the ruminal fluid of Saanen goats [9]. A study with dairy cows [10] demonstrated that incorporating a blend of tuna fish oil and linseed oil in the diet increased CLA, ALA, and DHA simultaneously in milk. To the best of our knowledge, no published research has evaluated the probable effect of combining fish oil and linseed oil on milk yield, composition, and milk FA profiles in dairy goats. The main objective of this research was to examine how milk production, composition, and FA profiles were affected by the supplementation of linseed oil alone or in combination with fish oil. We hypothesized that supplementing a combination of linseed oil and fish oil would increase health-promoting FA in milk with minimal effects on intake and ruminal fermentation.

## 2. Materials and Methods

The study was performed at Experimental Farm, Can Tho University, Vietnam. All procedures were carried out in compliance with the ethical standards stated in the Helsinki Declaration of 1975, revised in 2000, in addition to following the national laws.

### 2.1. Animals

Four F3 crossbred lactating Saanen goats (♂Saanen × ♀Bach Thao), mid-lactation, second parity, 1.30 ± 0.28 kg of milk and 36.6 ± 1.65 kg of body weight, were housed in individual wooden cages (1.2 m × 0.6 m × 1.2 m, L × W × H) and offered daily rations as equal meals at 7:00 and 17:00 h. The animals had free access to water and a mineral block and had enough space to exercise. Prior to conducting the experiment, goats had ad libitum access to a basal diet for one week to determine maximum feed intake.

### 2.2. Experimental Design and Diets

The animals were assigned to a 4 × 4 Latin square, each period consisting of 16 days for adjustment and 5 days for sampling. During the experimental period, all goats were fed a basal diet consisting of 40% fresh Para grass (*Brachiaria mutica*) and 60% pelleted concentrate (dry matter basis). Treatments were as follows: (1) basal diet without oil inclusion as a control (Ctrl) or the control plus 2.5% linseed oil (LO_2.5_), 2.5% linseed oil and tuna oil (3:2 w:w; LFO_2.5_), or 4.16% linseed oil and tuna oil (3:2 w:w; LFO_4.16_). The ratio (3:2 w:w) of linseed oil and tuna oil used was 2.5% DM, similar to a previous study [9]. The oil blend was added at 4.16% in the LFO_4.16_ diet such that this diet contained the same amount of linseed oil as the LO_2.5_ diet. The concentrate was mixed and pelleted once a week. Oil was mixed daily with concentrate before feeding. They then had ad libitum access to Para grass. Diets were monitored daily to ensure that the goats consumed exactly the ratios as they were designed. Feed ingredients and chemical composition of the diets are shown in Table 1 and Table 2.

### 2.3. Sampling and Measurements

Feed offered and refused was recorded daily for each goat during a five-day period (d15–d19). Feed samples were dried in a forced-air oven (FD 53, Binder, Tuttlingen, Germany) at 60 °C for 72 h. After this, the samples were stored at −20 °C until analyses of chemical composition.

Goats were milked daily at 7:30 and 17:30 h, and milk yields were recorded at each milking. Milk was sampled on two consecutive days (d19–d20) to analyze for major components. To measure milk FA composition, pooled milk samples from two-day samplings were stored at –20 °C until further analysis. To count somatic cells in milk, samples were taken twice (morning and afternoon) at the beginning and the end of each period.

On d21, ruminal fluid samples were collected at 0 and 3 h post morning feeding via stomach tube through the esophagus. Ruminal pH was immediately determined using a digital pH meter (HI-5522, Hanna Instruments, Inc., Woonsocket, Rhode Island, USA). The subsample was then filtered through a clean double-layer cotton cloth, and the liquid fraction was acidified with 1M H_2_SO_4_ (9:1 *v*/*v*), centrifuged at 10,000× *g* for 15 min, and stored at −20 °C until analyses of volatile fatty acids (VFA) and NH_3_-N concentrations.

### 2.4. Chemical Analysis

Samples were ground through a 1 mm mesh (Cutting Mill SM100, Retsch, Haan, Germany) and subjected to proximate analysis. Feed samples were analyzed for dry matter (DM), organic matter (OM), crude protein (CP), crude fiber (CF), ether extract (EE), and ash using standard methods [11]. Analyses of neutral detergent fiber (NDF) and acid detergent fiber (ADF) were done using methods of [12]. The concentration of NH_3_-N was analyzed by the micro-Kjeldahl method. Milk composition, including total solids, lactose, protein, fat and solids-not-fat, was analyzed with a MilkoScan infrared automatic analyzer (MilkoScan Mars, Foss, Hilleroed, Denmark). Milk samples were warmed at 40 °C in a shaking incubator (ISS-4075R, Jeiotech, Daejeon, Republic of Korea) prior to the analysis of milk composition. Milk samples were kept in an Eppendorf tube at 1 °C and immediately analyzed for somatic cells using a milk somatic cell analyzer (Adam-SCC, Nano Entek Inc., Seoul, Republic of Korea).

Concentrations of individual VFA were analyzed using a Thermo Trace 1310 GC system (Thermo Scientific, Waltham, MA, USA) equipped with a flame ionization detector. Aliquots (1 μL) were injected at a split ratio of 10:1 into a 30 m × 0.25 mm × 0.25 μm Nukol fused-silica capillary column (Cat. No: 24107, Supelco, Sigma–Aldrich, St. Louis, MO, USA) with helium carrier gas set to a flow rate of 1 mL/min. The temperature program of the GC was set up following a published method [13]. Individual VFA peaks were identified based on their retention times, compared with external standards, including acetic, propionic, butyric, valeric, iso-butyric, and iso-valeric acids (Sigma–Aldrich, USA).

Lipids in feed samples (1 g) were extracted in a chloroform:methanol solution (2:1 v:v) following the traditional Folch procedure [14], with minor modifications as described previously [15]. Lipids in milk samples (2 mL) were extracted with 25% ammonium solution, 95% ethanol, diethyl ether, and petroleum ether, according to the method of Chouinard et al. (1997) [16]. One mL of internal standard (1 mg C13:0/mL chloroform) was added to all extracted lipids and evaporated to complete dryness under a N_2_ stream. Dried lipids of feed and milk samples were then methylated with 3 mL NaOH in methanol (0.5 M) followed by 2 mL of acetyl chloride in methanol (1:5 vol/vol). The FA methyl esters (FAME) were extracted twice with 2 mL of hexane, and pooled extracts were evaporated under a N_2_ stream until dryness. The residue was dissolved in 1 mL of hexane and analyzed using a gas chromatograph (Thermo Scientific Trace 1310 GC system, Waltham, MA, USA) equipped with a flame ionization detector. Aliquots (1 μL) were injected at a split ratio of 50:1 into a 100 m × 0.25 mm × 0.25 μm high polar fused silica capillary column (Cat. No: 24056, Supelco Inc., Bellefonte, PA, USA) with helium carrier gas set to a flow rate of 1 mL/min. The temperature program of the GC was set up according to a published method [17]. Individual FAME was identified by comparison of retention times with 37 components of a commercial standard FAME mix (Cat. No: CRM47885, Supelco Inc., Bellefonte, PA, USA), CLA mix (Cat. No: O5632, Sigma-Aldrich, Louis, MO, USA), and C18:1 *t*11 standard (Cat. No: CRM46905, Supelco Inc., Bellefonte, PA, USA).

### 2.5. Calculations

Metabolizable energy intake (ME) was calculated following a published equation [18]. Atherogenicity index = [12:0 + 4(14:0) + 16:0]/[MUFA + PUFA] and thrombogenicity index = (14:0 + 16:0 + 18:0)/[(MUFA + *n*-6 PUFA)/2 + 3(*n*-3 PUFA) + (*n*-3 PUFA/*n*-6 PUFA)] [19].

### 2.6. Statistical Analysis

Data were analyzed using GLM in trình SAS OnDemand for Academics 2021 (SAS Institute Inc., Cary, NC, USA) for a Latin square design with the statistical model Y*ijkl* = µ + A*i* + D*j* + P*k* + ε*ijkl*, where Y*ijkl* = the dependent variable, A*i* = the effect of animal (*i* = 4 levels), D*j* = the effect of diet (*j* = 4 levels), P*k* = the effect of period (*k* = 4 levels), and ε*ijkl* = the residual effect. Significant differences among treatment means were compared using the Tukey test. Statistical tests were performed using SAS OnDemand for Academics 2021 (SAS Institute Inc., Cary, NC, USA). A significant effect of treatment was declared at *p* < 0.05, and tendencies were declared at 0.05 ≤ *p* < 0.10.

## 3. Results

### 3.1. Intake

Diet had no effect on the intake of DM, CP, and ME (Table 3). Except for C12:0, C16:0, C18:0, and C18:2 *c*9,*c*12, the inclusion of oil resulted in a greater intake of all FA (Table 3). As expected, compared with other diets, the addition of LO alone or in combination with FO at 4.16% resulted in greater consumption of C18:3*n*-3 (21.2 g/d and 23.4 g/d; *p* < 0.05), which is the predominant FA in linseed oil (4.40–14.6 g/d). Intakes of C20:5*n*-3 and C22:6*n*-3 were greatest with LFO_4.16_ (3.49 g/d and 1.73 g/d) compared with LFO_2.5_ (1.88 g/d and 0.93 g/d) and other diets (undetected). The total FA intake with LFO_4.16_ was 2.52 and 1.42 times greater (*p* < 0.05) than those in the Ctrl and other diets with added oil, respectively.

### 3.2. Milk Yield and Composition

The milk yield of the experimental goats ranged from 1.34 to 1.44 kg/day and did not differ among the diets (*p* > 0.05; Table 4). The milk composition and somatic cell count remained unchanged (*p* > 0.05) regardless of source and level of oil inclusion in the diet.

### 3.3. Ruminal Fermentation Patterns

Compared with the Ctrl goats fed the basal diet without oil inclusion, there was no change in the percentage of individual VFA in goats fed with 4.16% oil (*p* > 0.05; Table 5). Compared with prior to feeding, total VFA concentration was higher at 3 h post-feeding (59.8–69.1 vs. 53.1–57.8 mM). Compared with the Ctrl goats fed the basal diet without oil inclusion, there was no change in the percentage of individual VFA in goats fed 4.16% oil (*p* > 0.05; Table 5).

### 3.4. Milk Fatty Acids

Supplementation of oils altered the proportions of some medium- and long-chain SFA in milk, including C10:0, C11:0, C12:0, and C14:0 (Table 6). Compared with the Ctrl group (9.30 and 0.24%, respectively), goats fed LFO_4.16_ had the lowest (*p* < 0.05) concentrations of C10:0 and C11:0 (5.75 and 0.14%, respectively). The inclusion of oil at 2.5% led to a lower concentration of C12:0 (3.16–3.18%) and C14:0 (11.0–11.4%), but a greater (*p* < 0.001) concentration of these FA in the LFO_4.16_ (2.25 and 8.59%) compared with Ctrl diet (4.45 and 15.2%). As expected, the concentrations of beneficial FA, including C18:1 *t*11, C18:1 *c*9, *c*9,*t*11 CLA, and C22:6*n*-3 were markedly greater (*p* < 0.05; Table 6) in goats receiving LFO_4.16_. Supplementing the combination of LO and FO at 4.16% significantly increased C18:1 *t*11 (5.65%) compared with the Ctrl and SO added alone, respectively (0.82 and 1.40%; *p* < 0.01). Compared with Ctrl and LO_2.5_ diets, milk *c*9,*t*11 CLA was 4.53 and 2.94 times, respectively, significantly greater (*p* < 0.01) with the LFO_4.16_ diet. Compared with the Ctrl and LO_2.5_ diets (0.06% and 0.08%), goats fed LFO_2.5,_ and LFO_4.16_ had greater (*p* < 0.001) concentrations of C22:6*n*-3 (0.63% and 0.87%).

Compared with other diets, feeding LO alone or in combination with FO at 4.16% led to markedly greater (*p* < 0.01; Table 7) concentrations of C18 UFA. Feeding LO_2.5_ and LFO_4.16_ led to lower SFA and greater UFA, especially MUFA, in milk fat (*p* < 0.01; Table 7). As expected, PUFA were greatest (*p* < 0.05) in LFO_4.16_ (6.39%) compared with lower values (2.64% and 3.70%) detected in the Ctrl and LO_2.5_. Additionally, the percentage of total CLA increased (*p* < 0.01) from 0.54% to 0.81% with the Ctrl and LO_2.5_ diets to 2.43% in the LFO_4.16_. Goats fed the LFO_4.16_ diet exhibited a tendency for greater (*p* = 0.062) MUFA/SFA and greater (*p* < 0.05) PUFA/SFA in comparison with those fed the Ctrl diet; as the result of lower proportions of SFA and greater proportions of MUFA and PUFA with the LFO_4.16_ diet, atherogenicity and thrombogenicity indices in milk fat decreased by 2.09- and 1.69-fold (*p* < 0.05; Table 7) relative to the Ctrl diet. Yields of *c*9,*t*11 CLA and *n*-3 PUFA in milk fat were greater (*p* < 0.05) in the LFO_4.16_ group compared with those in Ctrl and LO_2.5_ groups (Table 8). In contrast, compared with the Ctrl group, the transfer of C18:2 *c*9,*c*12, C18:3*n*-3, and *n*-3 PUFA from feed to milk fat was lower (*p* < 0.05) in other groups (Table 8).

## 4. Discussion

### 4.1. Intake

The present study detected no significant impact of oil supplementation on total dry matter intake, which was in line with the findings of some earlier studies in which dairy goats were fed diets containing 3% DM linseed oil [7] and 2.5% soybean oil [20]. In a recent study conducted with dairy cows, total intake tended to decrease when animals were fed a 3% mixture of linseed, sunflower, and tuna oil [6]. The inclusion of either 2.2% FO or 5.3% sunflower oil in the diet had no effect on total DM intake in dairy goats but decreased total DM intake in dairy cows [21]. These findings revealed that dairy goats have a lower sensitivity toward reduction in intake in the presence of oils added to the diet. These types of responses led to a recommendation to limit total fat intake in the diet to 6–7% DM, as higher concentrations may result in a decrease in DM intake [22]. In the present study, the highest concentration of EE in the diets was 6.56%.

### 4.2. Milk Yield and Composition

The goats used in this research had a relatively low body weight and moderate milk production, which could be attributed to the tropical conditions they were managed in. When the air temperature, temperature–humidity index, and rectal temperature exceed certain critical thresholds, a decline in DM intake and a decrease in milk yield occur in tropical ruminants. Additionally, this can lead to a reduction in the efficiency of milk yield [23].

There have been inconsistent results in ruminants-fed oil supplements. Milk fat depression (MFD) was reported when oils were supplemented to cows [6,24,25], but milk fat content remained unchanged when cows were fed 4% linseed oil [26,27], and goats were fed a 2.5% oil blend [19]. The negative impact of adding oil to the diet of dairy ruminants, which leads to a reduction in milk fat content, is more commonly detected when the lipid sources used are high in PUFA.

In most of the experimental conditions that have been studied, MFD is partially linked to a change in ruminal biohydrogenation. This leads to the production of various ruminal intermediates such as *t*10 18:1 and *t*10,*c*12 CLA, which may adversely affect the mRNA abundance of lipogenic enzymes [28]. When dairy cows were fed milk fat-depressing diets, it resulted in the inhibition of mRNA abundance of mammary lipogenic enzymes. Additionally, supraphysiological concentrations of *t*10,*c*12 CLA were originally associated with MFD [29]. In the current study, the milk fat content remained unchanged despite the detection of higher concentrations of *t*10,*c*12 CLA in the LFO_4.16_ diet. It is worth noting that goats are less responsive to fat supplements compared with cows [30], which may explain the lack of differences in milk composition observed in this study. Milk somatic cell counts for goats in this study fluctuated within the standard range for goat milk, which is 1000 × 10^3^/mL [31], ranging from 658 to 1032 × 10^3^/mL at the beginning and from 435 to 1046 × 10^3^/mL at the end of the experiment.

### 4.3. Ruminal Fermentation Patterns

In this study, we found no effect of oil addition on ruminal fermentation patterns. However, a study in dairy cows [6] reported that oil inclusion at 3% in the diet reduced total ruminal VFA concentration. Dairy goats fed linseed oil at 20 mL/day (approximately 2.3% of DM) had greater total ruminal VFA concentrations [32]. Inconsistent results may be attributed to differences in species, stage of lactation, forage sources in the diet, and forage-to-concentrate ratio. Compared with dairy cows, the addition of vegetable oil to the diet of dairy goats had a modest negative effect [33].

### 4.4. Milk Fatty Acid Composition

The reduction in C10:0–C14:0 content (*p* < 0.01) was partially attributed to slight alterations in the activity of acetyl-CoA carboxylase and other enzymes that are involved in the de novo synthesis of SFA in the mammary gland [34]. The reduction in C12:0 and C14:0 concentrations in milk fat from goats supplemented with linseed oil and fish oil could have a favorable impact on human health since the consumption of these FA was reported to have an inverse correlation with the occurrence of heart attacks [35].

Greater milk *c*9,*t*11 CLA in goats fed LFO_4.16_ was in agreement with a previous finding [36]. This FA originates from the biohydrogenation of linoleic acid and α-linolenic acid in the rumen as an intermediate or from the endogenous synthesis in the mammary gland from vaccenic acid [37]. In this study, the goats supplemented with extruded linseed and fish oils had a greater DHA content in milk (0.63–0.87 g/100 g FA) compared with previous results (0.098 g/100 g FA) [38] when goats were supplemented with extruded linseed and fish oil. Consumption of dairy products that have lower atherogenic index values, such as DHA, results in a reduction of total cholesterol in the human plasma [39].

The current research was designed to include linseed oil and fish oil at 4.16%, resulting in an enhancement of milk concentrations of MUFA and PUFA. These findings align with previous studies [6,40]. In this study, the inclusion of a high PUFA oil mixture in the diet led to a decrease in the atherogenicity index and thrombogenicity index, which can effectively counteract the negative impact of high levels of SFA and *n*-6 FA present in milk. A notable decrease in atherogenicity and thrombogenicity indices was also detected when cows were fed 3% linseed oil and fish oil [10] or 3% of linseed oil, sunflower oil, and fish oil [6]. The lower transfer efficiency of C18:2 *c*9,*c*12, C18:3*n*-3, and *n*-3 PUFA in the goats fed oil inclusion was in agreement with a previous study [10] where a lower apparent transfer in the diets was observed in diets containing higher PUFA concentration.

A limitation of this study was that the sensory characteristics of milk were not measured; therefore, it could not be determined whether the inclusion of fish oil affected the sensory quality of milk. It was shown in a published paper that no significant difference was detected in the flavor characteristics of milk and butter from cows fed the control (without fish oil inclusion) and fish oil diets [41]. Moreover, supplementation of 3% fish oil in the dairy cow diet did not have any detrimental effects on ice cream’s physicochemical and sensory characteristics [42].

## 5. Conclusions

There was no impact on intake, ruminal fermentation patterns, milk yield, and milk composition when linseed oil and fish oil were incorporated at 4.16% in the diet of lactating goats. However, this diet effectively increased the levels of health-promoting fatty acids in milk, such as C18:1 *t*11, *c*9,*t*11 CLA, and C22:6*n*-3. Additionally, it decreased milk total SFA, atherogenicity, and thrombogenicity indexes. Thus, supplementing linseed oil and fish oil at 4.16% in the diet of lactating goats could have a positive impact on human health without any adverse effect on animal performance.

## Figures and Tables

**Table 1:** Ingredients and chemical composition of experimental diets.

Item	Diet ^1^			
	Ctrl	LO_2.5_	LFO_2.5_	LFO_4.16_
Ingredient, % DM				
Soybean meal	15.2	15.7	15.6	15.9
Ground corn	15.5	9.46	9.03	-
Rice bran	6.85	11.45	12.0	20.2
Para grass	60.0	58.5	58.5	57.5
NaCl	0.30	0.30	0.30	0.30
Premix ^2^	0.50	0.50	0.50	0.50
CaCO_3_	1.74	1.62	1.53	1.43
Linseed oil	-	2.50	1.50	2.50
Tuna fish oil	-	-	1.00	1.66
Chemical composition, % of DM unless otherwise noted				
DM	45.6	46.7	46.7	47.8
Ash	9.80	10.2	10.2	11.2
OM	90.2	89.8	89.8	88.8
CP	17.9	17.9	17.9	18.3
NDF	46.8	50.7	50.7	50.1
ADF	25.4	26.4	26.4	29.4
CF	23.2	23.9	23.9	25.9
NFE	46.9	43.3	43.3	38.4
EE	2.23	4.74	4.74	6.56
ME, Mcal/kg DM	2.33	2.55	2.55	2.61

DM: dry matter, Ash: total minerals, OM: organic matter, CP: crude protein, NDF: neutral detergent fiber, ADF: acid detergent fiber, CF: crude fiber, NFE: nitrogen-free extract, EE: ether extract, ME: metabolizable energy. ^1^ Ctrl: control; LO_2.5_: 2.5% linseed oil; LFO_2.5_: 2.5% linseed oil + fish oil (3:2 wt:wt); LFO_4.16_: 4.16% linseed oil + fish oil (3:2 wt:wt). ^2^ Content in 1 kg premix included: 290–350 g Ca, P: 62, 35 mg Mg, 450,000 UI vitamin A, 70,000 UI vitamin D, 1800 UI vitamin E, and others.

**Table 2:** Fatty acid composition of the diet.

Fatty Acid (g/100 g FA)	Feed				Diet ^1^			
	Fish Oil	Linseed Oil	Para Grass	Concentrate	Ctrl	LO_2.5_	LFO_2.5_	LFO_4.16_
C12:0	0.08	0.01	0.69	0.03	0.43	0.42	0.42	0.42
C14:0	6.33	0.06	0.84	0.17	0.57	0.55	0.63	0.70
C16:0	21.6	5.52	47.2	19.5	36.1	38.3	38.3	39.1
C18:0	2.03	3.22	10.1	5.34	8.20	8.04	8.67	7.54
C18:1 *c*9	13.9	17.9	3.63	35.6	16.4	18.7	18.1	20.2
C18:2 *c*9,*c*12	2.39	16.5	11.4	26.1	17.3	16.6	16.3	13.9
C18:3*n*-3	0.29	55.8	21.5	0.68	13.2	14.2	13.7	14.1
C20:5*n*-3	14.9	nd ^2^	nd	nd	-	-	0.15	0.25
C22:6*n*-3	7.37	nd	nd	nd	-	-	0.07	0.12
SFA	45.2	9.06	62.3	37.0	52.2	49.7	50.6	50.3
UFA	54.8	90.9	37.7	63.0	47.8	50.3	49.4	49.7
MUFA	29.0	18.0	4.65	36.1	17.2	19.4	19.0	21.2
PUFA	25.8	72.9	33	26.9	30.6	30.9	30.3	28.5
*n*-3 PUFA	22.9	56.1	21.5	0.76	13.2	14.2	13.9	14.5
*n*-6 PUFA	2.75	16.5	11.5	26.1	17.3	16.7	16.4	14.0

^1^ Ctrl: control; LO_2.5_: 2.5% linseed oil; LFO_2.5_: 2.5% linseed oil + fish oil (3:2 wt:wt); LFO_4.16_: 4.16% linseed oil + fish oil (3:2 wt:wt). ^2^ nd: Not detectable.

**Table 3:** Intakes in dairy goats fed a basal diet without or with an added mixture of linseed oil and fish oil.

Item	Diet ^1^				SEM	*p*
	Ctrl	LO_2.5_	LFO_2.5_	LFO_4.16_		
Main components						
DM, g/day	1635	1517	1514	1414	112	0.190
CP, g/day	297	274	274	262	24.0	0.300
ME, Mcal/d	3.89	3.94	3.94	3.73	0.31	0.768
Fatty acids ^2^, g/d						
C12:0	0.14	0.14	0.14	0.14	0.01	0.896
C14:0	0.20 ^b^	0.20 ^b^	0.99 ^a^	1.67 ^a^	0.15	0.001
C16:0	12.7	13.5	15.5	18.2	1.24	0.074
C18:0	2.92	3.71	3.56	4.21	0.33	0.138
C18:1 *c*9	6.74 ^b^	11.8 ^ab^	11.4 ^ab^	16.4 ^a^	1.54	0.025
C18:2 *c*9,*c*12	6.68	11.3	9.6	12.8	1.26	0.059
C18:3*n*-3	4.40 ^b^	21.2 ^ab^	14.6 ^ab^	23.4 ^a^	3.29	0.024
C20:5*n*-3	n.d.	n.d.	1.88 ^b^	3.49 ^a^	0.35	0.001
C22:6*n*-3	n.d.	n.d.	0.93 ^b^	1.73 ^a^	0.17	0.001
SFA	18.7 ^b^	20.2 ^ab^	24.7 ^ab^	30.5 ^a^	2.16	0.030
UFA	18.2 ^b^	44.8 ^ab^	40.8 ^ab^	62.1 ^a^	6.58	0.018
MUFA	7.02 ^b^	12.1 ^ab^	13.6 ^ab^	20.2 ^a^	1.80	0.012
PUFA	11.1 ^b^	32.7 ^ab^	27.2 ^ab^	41.9 ^a^	4.83	0.021
*n*-3 PUFA	4.42 ^b^	21.3 ^ab^	17.5 ^ab^	28.8 ^a^	3.59	0.016
*n*-6 PUFA	6.70 ^b^	11.3 ^ab^	9.65 ^ab^	12.9 ^a^	1.26	0.058
Total FA	36.8 ^b^	65.1 ^ab^	65.5 ^ab^	92.7 ^b^	8.51	0.021

^1^ Ctrl: control; LO_2.5_: 2.5% linseed oil; LFO_2.5_: 2.5% linseed oil + fish oil (3:2 wt:wt); LFO_4.16_: 4.16% linseed oil + fish oil (3:2 wt:wt). ^2^ n.d. indicates proportions of FA in feed ingredients not detected or below 0.01% of total FA. ^a,b^ Means within a row with different superscripts are significantly different at *p* < 0.05.

**Table 4:** Milk yield and composition in dairy goats fed a basal without or with an added mixture of linseed oil and fish oil.

Item	Diet ^1^				SEM	*p*
	Ctrl	LO_2.5_	LFO_2.5_	LFO_4.16_		
Milk yield, kg/day	1.44	1.34	1.36	1.44	0.15	0.687
Milk composition, %						
Fat	2.78	3.18	2.83	3.03	0.57	0.743
Protein	3.08	3.02	3.04	2.92	0.12	0.363
Lactose	4.26	4.35	4.38	4.41	0.66	0.508
Solid not fat	8.14	8.18	7.71	7.96	0.42	0.451
Total solid	10.5	10.7	10.6	10.9	0.89	0.716
Somatic cell count, ×10^3^/mL						
Initial	1032	1007	715	658	538	0.696
Final	692	720	435	1046	357	0.221

^1^ Ctrl: control; LO_2.5_: 2.5% linseed oil; LFO_2.5_: 2.5% linseed oil + fish oil (3:2 wt:wt); LFO_4.16_: 4.16% linseed oil + fish oil (3:2 wt:wt).

**Table 5:** Ruminal fermentation patterns in dairy goats fed a basal diet without or with an added mixture of linseed oil and fish oil.

Item	Diet ^1^				SEM	*p*
	Ctrl	LO_2.5_	LFO_2.5_	LFO_4.16_		
0 h						
pH	6.80	6.90	6.88	6.81	0.10	0.490
NH_3_-N, mg/dL	32.2	37.8	31.5	30.1	4.50	0.182
Total VFA, mM	54.9	57.8	53.5	53.1	4.39	0.476
Acetate,%	67.0	66.4	65.3	64.7	1.62	0.254
Probionate, %	17.7	18.8	19.3	20.1	1.57	0.286
Acetate/propionate	3.78	3.54	3.38	3.22	0.38	0.254
Iso-butyrate, %	3.81	3.60	3.39	3.23	0.13	0.608
Butyrate, %	1.85	1.82	1.78	1.73	0.49	0.466
Iso-valerate, %	8.59	8.26	8.80	8.73	0.17	0.734
Valerate, %	2.53	2.55	2.52	2.43	0.17	0.572
3 h						
pH	6.52	6.56	6.63	6.74	0.12	0.128
NH_3_-N, mg/dL	37.1	32.6	37.8	29.4	5.35	0.190
Total VFA, mM	64.5	63.0	69.1	59.8	6.47	0.321
Acetate,%	66.7	66.4	67.1	66.0	2.66	0.941
Probionate, %	19.0	19.5	19.0	19.7	1.70	0.318
Acetate/propionate	3.50	3.40	3.53	3.34	0.71	0.953
Iso-butyrate, %	3.62	3.49	3.62	3.36	0.13	0.585
Butyrate, %	1.60	1.62	1.58	1.70	0.52	0.916
Iso-valerate, %	8.14	8.21	8.23	8.17	0.16	0.612
Valerate, %	2.19	2.22	2.16	2.31	0.15	0.403

^1^ Ctrl: control; LO_2.5_: 2.5% linseed oil; LFO_2.5_: 2.5% linseed oil + fish oil (3:2 wt:wt); LFO_4.16_: 4.16% linseed oil + fish oil (3:2 wt:wt).

**Table 6:** Milk individual fatty acid composition in dairy goats fed a basal diet without or with an added mixture of linseed oil and fish oil.

Fatty Acid (g/100 g FA)	Diet ^1^				SEM	*p*
	Ctrl	LO_2.5_	LFO_2.5_	LFO_4.16_		
Saturated FA						
C4:0	0.44	0.81	0.40	0.56	0.28	0.255
C6:0	1.30	1.74	1.14	1.35	0.39	0.255
C8:0	1.69	1.93	1.39	1.43	0.45	0.371
C10:0	9.30 ^a^	7.47 ^ab^	6.64 ^ab^	5.75 ^b^	1.16	0.024
C11:0	0.24 ^a^	0.21 ^ab^	0.17 ^ab^	0.14 ^b^	0.03	0.021
C12:0	4.45 ^a^	3.18 ^b^	3.16 ^b^	2.25 ^c^	0.23	<0.001
C14:0	15.2 ^a^	11.0 ^b^	11.4 ^b^	8.59 ^c^	0.66	<0.001
C15:0	0.99	0.96	1.08	1.07	0.32	0.499
C16:0	36.2	29.1	40.0	35.8	5.23	0.120
C17:0	0.66	0.59	0.68	0.65	0.08	0.472
C18:0	6.49	10.2	8.52	7.94	2.73	0.366
C20:0	0.03	0.04	0.09	0.12	0.05	0.106
C21:0	0.01	0.25	0.01	0.21	0.34	0.653
C22:0	0.03	0.04	0.09	0.12	0.05	0.105
C23:0	0.02	0.02	0.02	0.03	0.01	0.422
C24:0	0.02	0.02	0.03	0.04	0.01	0.096
Unsaturated FA						
C14:1	0.29	0.20	0.20	0.15	0.06	0.080
C15:1	0.01	0.14	0.00	0.12	0.20	0.667
C16:1	0.65	0.56	0.60	0.69	0.13	0.525
C17:1	0.06	0.05	0.05	0.06	0.08	0.990
C18:1 *t*9	0.07	0.14	0.20	1.99	1.74	0.406
C18:1 *t*11	0.82 ^b^	1.40 ^b^	2.70 ^ab^	5.65 ^a^	1.25	0.006
C18:1 *c*9	18.4 ^ab^	26.2 ^a^	16.8 ^b^	18.6 ^ab^	3.79	0.047
C18:2 *t*9,*t*12	0.12 ^b^	0.29 ^ab^	0.38 ^a^	0.50 ^a^	0.10	0.008
C18:2 *c*9,*c*12	1.13	1.58	1.07	1.30	0.21	0.054
*c*9,*t*11 CLA	0.51 ^b^	0.79 ^b^	1.38 ^ab^	2.32 ^a^	0.49	0.007
*c*12,*c*12 CLA	0.01	0.01	0.02	0.02	0.02	0.413
*t*10,*c*12 CLA	0.02 ^b^	0.02 ^b^	0.02 ^b^	0.09 ^a^	0.02	0.012
C18:3*n*-6	0.01	0.01	0.01	0.01	0.01	0.454
C18:3*n*-3	0.68	0.78	0.80	0.91	0.25	0.644
C20:1*n*-9	0.02 ^b^	0.02 ^b^	0.06 ^ab^	0.14 ^a^	0.04	0.021
C20:2	0.01	0.01	0.01	0.02	0.01	0.075
C20:3*n*-6	0.01	0.01	0.01	0.01	0.01	0.517
C20:3*n*-3	0.01	0.01	0.01	0.03	0.01	0.090
C20:4*n*-6	0.04	0.04	0.04	0.04	0.02	0.985
C20:5*n*-3	0.03	0.04	0.03	0.07	0.03	0.273
C22:1*n*-9	0.01	0.01	0.01	0.03	0.01	0.090
C22:2	0.01	0.02	0.09	0.19	0.16	0.421
C22:6*n*-3	0.06 ^b^	0.08 ^b^	0.63 ^a^	0.87 ^a^	0.07	<0.001
C24:1*n*-9	0.06	0.04	0.08	0.11	0.05	0.406

^1^ Ctrl: control; LO_2.5_: 2.5% linseed oil; LFO_2.5_: 2.5% linseed oil + fish oil (3:2 wt:wt); LFO_4.16_: 4.16% linseed oil + fish oil (3:2 wt:wt). ^a,b,c^ Means within a row with different superscripts are significantly different at *p* < 0.05.

**Table 7:** Milk fatty acid group composition in dairy goats fed a basal diet without or with an added mixture of linseed oil and fish oil.

Fatty Acid (g/100 g FA)	Diet ^1^				SEM	*p*
	Ctrl	LO_2.5_	LFO_2.5_	LFO_4.16_		
FA groups						
C18 UFA	21.7 ^b^	31.2 ^a^	23.4 ^b^	31.4 ^a^	0.94	0.001
SFA	77.0 ^a^	67.6 ^b^	74.8 ^a^	66.1 ^b^	2.25	0.001
UFA	23.0 ^b^	32.4 ^a^	25.2 ^b^	33.9 ^a^	2.25	0.001
MUFA	20.4 ^b^	28.7 ^a^	20.7 ^b^	27.5 ^a^	1.45	0.003
PUFA	2.64 ^b^	3.70 ^b^	4.48 ^ab^	6.39 ^a^	1.31	0.032
*n*-3 PUFA	0.78	0.91	1.47	1.89	0.74	0.229
*n*-6 PUFA	1.31	1.95	1.50	1.86	0.28	0.055
Total CLA	0.54 ^b^	0.81 ^b^	1.42 ^ab^	2.43 ^a^	0.25	0.007
Indices						
MUFA/SFA	1.13	1.13	1.22	1.23	0.03	0.062
PUFA/SFA	0.03 ^b^	0.05 ^ab^	0.06 ^ab^	0.09 ^a^	0.01	0.027
Atherogenecity index	4.61 ^a^	2.37 ^b^	3.59 ^ab^	2.21 ^b^	0.72	0.010
Thrombogenicity index	4.32 ^a^	2.74 ^ab^	3.72 ^ab^	2.56 ^b^	0.71	0.038

^1^ Ctrl: control; LO_2.5_: 2.5% linseed oil; LFO_2.5_: 2.5% linseed oil + fish oil (3:2 wt:wt); LFO_4.16_: 4.16% linseed oil + fish oil (3:2 wt:wt). ^a,b^ Means within a row with different superscripts are significantly different at *p* < 0.05.

**Table 8:** Milk fatty acids secreted relative to corresponding dietary fatty acids.

Item	Diet ^1^				SEM	*p*
	Ctrl	LO_2.5_	LFO_2.5_	LFO_4.16_		
Yield of milk fatty acids (g/d)						
C18:2 *c*9,*c*12	0.43	0.65	0.38	0.53	0.07	0.153
C18:3*n*-3	0.25	0.27	0.28	0.34	0.05	0.632
*c*9,*t*11 CLA	0.20 ^b^	0.31 ^b^	0.55 ^ab^	0.98 ^a^	0.11	0.011
*n*-3 PUFA	0.29 ^b^	0.31 ^b^	0.53 ^ab^	0.75 ^a^	0.09	0.026
*n*-6 PUFA	0.50	0.80	0.54	0.76	0.09	0.164
Transfer into milk (g/100 g FA intake)						
C18:2 *c*9,*c*12	5.94 ^a^	4.90 ^ab^	3.24 ^b^	3.50 ^ab^	0.55	0.039
C18:3*n*-3	6.15 ^a^	1.04 ^b^	1.71 ^b^	1.48 ^b^	0.64	0.004
*c*9,*t*11 CLA	1.77	0.83	1.86	2.50	0.48	0.201
*n*-3 PUFA	7.08 ^a^	1.20 ^b^	2.59 ^ab^	2.59 ^ab^	0.92	0.018
*n*-6 PUFA	6.91	6.07	4.55	5.00	0.69	0.164

^1^ Ctrl: control; LO_2.5_: 2.5% linseed oil; LFO_2.5_: 2.5% linseed oil + fish oil (3:2 wt:wt); LFO_4.16_: 4.16% linseed oil + fish oil (3:2 wt:wt). ^a,b^ Means within a row with different superscripts are significantly different at *p* < 0.05.

## Data Availability

The data presented in this study are available upon reasonable request from the corresponding author.
